# Comparison of coverage with insecticide-treated nets in a Tanzanian town and villages where nets and insecticide are either marketed or provided free of charge

**DOI:** 10.1186/1475-2875-5-44

**Published:** 2006-05-21

**Authors:** CA Maxwell, RT Rwegoshora, SM Magesa, CF Curtis

**Affiliations:** 1 London School of Hygiene & Tropical Medicine, London WC1E 7HT, UK; 2 National Institute for Medical Research, Amani Medical Research Centre, Box 81, Muheza, Tanzania

## Abstract

**Background:**

There is much emphasis on social marketing as a means of scaling up coverage with insecticide-treated nets and the question has arisen whether nets provided free-of-charge will be looked after by householders.

**Methods:**

Over several years questionnaires and surveys of usage and condition of nets were carried out throughout a town and 15 villages in north-east Tanzania, where nets and insecticide have to be purchased and in 24 other villages where over 15000 nets had been donated and annual re-treatment is provided free-of-charge.

**Results:**

There was very high population coverage in the town but, in the villages where nets have to be purchased, only 9.3% of people used nets which were intact and/or had been insecticide-treated and could, therefore, provide protection. However, where nets had been provided free, over 90% of the nets were still present and were brought for re-treatment several years later.

**Conclusion:**

In this part of Tanzania, social marketing has performed well in a town but very poorly in villages. However, the study showed that people look after and bring for re-treatment nets which had been provided free-of-charge.

## Background

Bednets provide no protection if torn and untreated but, if intact and/or insecticidal, nets provide good, but not perfect, personal protection to sleepers against night biting mosquitoes [1-3]. If used by almost all members of a community, insecticidal nets kill large numbers of the local malaria vectors, reducing the mean survival, sporozoite rate and population density of the vector population and hence substantially reducing its entomological inoculation rate (EIR) [4]. This "bonus" effect of community-wide coverage of insecticide treated nets (ITNs) can equal or exceed the personal protection effect [5,6].

Especially in Tanzania, there has been much emphasis on scaling up coverage with ITNs via social marketing and, comparing households who were able and willing to purchase nets with those who were not, there was significantly less child mortality and malaria morbidity in the former [7,8]. The argument advanced in favour of social marketing is that sustained donor funding to provide ITNs free of charge cannot be relied upon. It is therefore considered preferable to use existing donor funding to subsidize schemes which use advertising to encourage householders to become accustomed to spending their own money to buy nets and insecticide.

In assessing whether marketing schemes are well targeted and effective nationwide it is necessary to take account of the marked social and entomological heterogeneities between urban and rural areas. In the former, most households are supported by wage earners and people are much troubled by the nuisance biting of *Culex *mosquitoes which breed in organically polluted water in pit latrines, cess pits and open drains. There are generally few breeding sites suitable for *Anopheles *and malaria transmission is much lower in long established and densely populated urban areas, in contrast to nearby rural or semi-rural areas [9]. In African villages many households are subsistence farmers with relatively little involvement in a cash economy and the ground water is generally clean enough for the breeding of large numbers of *Anopheles gambiae s.l*.. and *Anopheles funestus*. Therefore, these communities suffer the majority of Africa's and the world's malaria burden.

In assessing the cost to donors of a sustained scheme for free provision of ITNs it is necessary to assess how well donated nets are looked and after how many years nets would have to be replaced because they have become so torn that householders discard them Hitherto there has been little data available on these factors.

## Methods and materials

In the course of censuses in 2001–2005, in preparation for proposed future multi-village trials with ITNs, all householders were questioned in 15 villages in Muheza district, north east Tanzania, about which members of the family used nets and whether they had been insecticide-treated. In total, data were collected on net usage by 16683 people. The nets were also inspected to determine whether they were intact or holed, according to the definition of Maxwell *et al *[10]. In 2004, all houses in Muheza town were censused and householders were questioned about the total numbers of nets owned by the household and whether they had been treated. Data were collected on net usage by 4373 people. These urban nets were not inspected for damage, because the townspeople were less tolerant of intrusion into their houses than were the villagers. Thirdly, in 2001–2004 villages were re-visited in which, as far as possible, every sleeping place in every house had been provided with an indelibly numbered ITN free of charge against a householder's signature. The nets provided were made of 70 denier fibre. The distributions had been in the course of trials in eight lowland (altitude about 200 m) villages which started in 1995–6 [5,10] and 10 lowland and 11 highland (altitude approximately 1,000 m) villages starting in 2000 [6]. The total numbers of nets which had originally been provided in these three sets of villages were over 15000. Records were kept of nets being brought for the annual re-treatment, which is provided by a supervisor, visiting by motorcycle and bringing free insecticide. The nets being brought by each householder were matched with records of those previously given to that household, noting nets which had gone missing and recording the number of the nets which were intact or holed, as defined by Maxwell *et al *[10]. In some cases it was possible to repeat surveys in the same villages at different times after provision of nets but in other cases the surveys were in different villages in which human behaviour regarding their nets may vary somewhat. This was probably the reason for some apparently anomalous results such as somewhat more nets being recorded as intact after 6–7 years than after 5–6 years.

## Results and discussion

Table 1 shows that in Muheza town 93.2% of households had bought at least some nets and the total number of nets was about 50% of the human population revealed by our census, implying very high coverage of the population by nets, considering that many of the nets would be used by two or more people. It was reported that 27.2% of nets were insecticidal, i.e. had been treated at some time with an *Ngao *insecticide sachet (or in a few cases there were Permanet^® ^Long Lasting Insecticidal nets). The high net usage can mainly be attributed to a desire to avoid *Culex *nuisance biting and to response to advertising (urban billboards etc) organized by the Social Marketing campaign and net manufacturers. Appreciable numbers of *Anopheles *probably fly into a small town like Muheza from nearby semi-rural breeding sites and the nets presumably serve a useful anti-malaria function. The insecticide sachets are advertised in the town but uptake was not as good as for nets. The treated nets would have given personal protection even if holed. However, when only about a quarter of net encounters by *Anopheles *would lead to a risk of mortality, any consequent reduction in EIR of the local vector population would be far less than the EIR reductions observed when ITN coverage is so high that almost every net encounter by an *Anopheles *mosquito has a high probability of killing it. [4,6]. About half of the nets were of the conical type, with which it is rather difficult for a sleeper to avoid skin contact and there is some evidence [2] that these nets are not so effective as rectangular nets in preventing mosquitoes biting through nets.

**Table 1:** Data on the households in Muheza town

No. people surveyed by questionnaire:	4373
No. nets in their houses	2197 (approx. 0.5× no. people)
% of households reported to be using nets	93.2% (data on 948 households)
% of nets treated with *Ngao *insecticide sachets (+Permanets)	26.3% *Ngao*; 0.9% Permanets (data on 2197 nets)
% of nets rectangular (not conical)	45.6% (data on 2197 nets)

Table 2 shows that, compared with the urban data, there was an entirely different picture in those surveyed villages in which (as in the town) netting and insecticide had to be purchased by householders. The percentage of people reported to be using a net varied between villages but averaged only 16.9% over all age groups, with a somewhat higher rate among young children (who are the most vulnerable to malaria) and a somewhat lower rate among older children. The 2,824 people reported to be using a net and the total of 1,407 nets recorded imply a mean of 2.0 people per net (appreciably more than the mean of 1.7 found by Curtis *et al *[11]. There was no sign of a hoped-for upward trend in net usage over the years 2001–2005 as the social marketing programme developed and supposedly became nationwide in Tanzania. Inspections showed that only 51.1% of nets were intact and 10.6% of nets were reported to have been treated. 44.9% of nets were holed and untreated and therefore gave no protection; thus, only 9.3% of the population received any protection by nets from the intense malaria transmission in these villages.

**Table 2:** Data from censuses of 15 villages where nets and insecticide have to be bought

Questionnaires about who uses a net:-		
Year	% of people stated to be using a net (no. of people surveyed)	Range of % coverage in different villages (no. of villages surveyed)

2001	25.0% (1237)	25.0% (1)
2002	14.0% (5674)	10.7–26.9% (4)
2003	12.8% (2455)	7.8–24.5% (4)
2004	14.4% (3636)	4.7–20.0% (3)
2005	23.9% (3681)	20.9–28.0% (3)
All 5 years	16.9% (16683)	4.7–28.0% (15)
% reported net coverage in different age groups (no. people surveyed)-		
<6 yrs	22.2% (2684)	
6–12 yrs	14.1% (3272)	
>12 yrs	16.4% (10734)	
Observations on nets:-		
Total no. in the 15 villages	1407 (approx. 0.5× the 2824 people stated to be using a net, i.e average of about 2 people per net)	
% of nets intact*	51.1% (data on 1220 nets)	
% of nets stated to have been treated	10.6% (data on 1220 nets)	
% torn and untreated (i.e. giving no effective protection)	44.9% (data on 1220 nets)	

* by definition of Maxwell et al [10]

Table 3 shows that, for several years after provision of free nets in villages, about 90% were still in the households to which they were given and were brought for the annual re-treatment. In later years, in the lowlands, the retention of nets declined markedly. This was associated with decrease in the percentage of nets still intact. This indicates that householders understandably did not retain nets when they were so badly holed as to be obviously useless. After about 5 years the condition of the nets was markedly better in the highlands than the lowlands, presumably because in the cool season in the highlands there are few nuisance mosquitoes and many nets are not used and are, therefore, not exposed to wear and tear at that season. In the earlier years in the lowlands and highlands the mean numbers of nets per household were 1.9 and 2.3, respectively, with 4 or more in some households with many children.

**Table 3:** Data from surveys of nets still in houses and/or brought for re-treatment and % still intact in lowland (c.200 m) and highland (c.1000 m) villages where 70 denier nets had been provided free of charge for every bed several years before the surveys, and where re-treatment is provided annually

	Lowland			Highland	
	
	Nos. of years after nets were provided:-				
	
	3–4	5–6	6–7	2	4–5
Nos. of nets originally provided to surveyed house-holds (no. villages)	5238 (6)	5976 (8)	5493 (8)	5023 (10)	4842 (9)
% observed still in houses &/or brought for the annual re-treatment	91.3%	70.6%	59.6%	89.2%	91.7%
% of nets intact*	56.5%	32.8%	38.4%	98.3%	84.8%

*by definition of Maxwell et al [10]

It might be thought that provision of free nets to some villages in Muheza district would be well known to people in other villages and to have deterred sales there, as the inhabitants might have expected that eventually free nets would be given to them also. However, many lengthy conversations in the ki-Swahili language showed that in fact people in villages which had not been provided with free nets were not aware of the good fortune of the people in some other villages several kilometres away. It might also be supposed that the people in the "free net" project villages were under close supervision of project staff and, therefore, used and treated nets in a way quite atypical of what would happen if large scale free provision became a routine. However, with a team of only five field personnel involved in a number of different projects, contacts with villagers since malaria morbidity surveys were finished some years ago were virtually limited to the annual two to three days of net re-treatment in each of the villages when a technician visits to bring insecticide and supervises the re-treatment process. Such visits involve helpful contact and conversation about ITNs for malaria control and would be expected in a region-wide re-treatment programme, as exists in Vietnam where the nets of 10 million people are re-treated annually (see data of Tran Duc Hinh reproduced in [12]). Such a re-treatment scheme would become unnecessary if there was a complete switch over to use of long-lasting insecticidal nets. Such a switch over could be decided upon by a donor, based on evidence that it would be more cost effective over a four [13] or perhaps a seven-year period [14] to pay more for a long-lasting insecticidal net so as to avoid the expense of annual re-treatment. However, where a free market prevails it seems likely that many low income households would go for the net with the cheapest price tag, even if it would be wiser in the long run to invest in a more expensive long lasting insecticidal net. As shown in Table 1, less than 1% of the Muheza urban households had bought such long lasting nets.

In attempting to market nets to very poor people, much effort and expenditure is required on promotion. However, experience in this part of Tanzania shows that villagers are well aware of bednets and their benefits and the only problem about acquiring nets is finding the money to pay for them. If news is passed through the village authorities that the numbers and sizes of beds would be observed and appropriate ITNs provided free of charge on a certain day, virtually every household is waiting to enthusiastically receive and use these nets.

The estimates of Curtis et al [15] about the annual cost of continent-wide free provision of nets only considered rural populations because these bear the main burden of malaria. Furthermore it was surmised that marketing would work well in towns; this is confirmed by the data in Table 1. Higher altitudes with very low malaria transmission could also be excluded from schemes for free provision, but excellent impact of ITN provision on malaria morbidity at about 1,000 m [6] suggests that the cut-off altitude should be set somewhat higher than that. The above mentioned estimates [15] assumed that polyester nets would become so badly torn after about 4 years that they ought to be replaced then. The data in Table 3 confirm this for lowland villages, but it appears that for cooler highland areas less frequent replacement of nets would be acceptable. It is regrettable that, for the lowland villages referred to in Table 3, there was delay in finding the funding to replace the damaged nets. However, the nets have now been replaced in many of these villages in setting up a comparative trial of different types of long lasting insecticidal net. Worn out ITNs are being replaced in other villages through the generosity of Rotary Clubs.

The data in Table 3, showing the encouraging extent to which nets provided free of charge are retained and looked after, come from annual visits by a re-treatment supervisor to 24 villages. Now that millions of nets are being provided free-of-charge, for example in Eritrea, Togo and Niger, it will be important in the next few years to check whether or not our data based on 24 villages gave reliable indications of the long term retention and condition of nets in region- or nation-wide schemes.

In the Kilombero valley of Tanzania, where social marketing was initiated several years ago, surveys[16] found that a large proportion of the nets were badly torn and untreated with insecticide (and, therefore, unprotective) as was also found in the present study (Table 2). It is understood that some other net surveys in Tanzania have shown better coverage with ITNs in some rural areas and it will be important to determine whether the very poor results of marketing in villages shown in Table 2 are, or are not, typical. In making national assessments it is very important to be sure that samples are not biased in favour of easier-to-reach urbanized areas which may well have a quite atypically high net coverage (Table 1). In view of the mean of about 2 nets per household which was found where nets had earlier been provided free of charge, it should be noted that expressing national survey results as percentage of houses with at least one net could give a misleading impression of whether adequate coverage was being achieved.

The introduction of a voucher scheme to enable women attending ante-natal clinics to purchase nets at a subsidized price and linkage of provision of free ITNs to attendance of children at measles vaccination campaigns [17] will hopefully greatly improve rates of personal protection of the most malaria vulnerable members of the population. However, it is doubtful whether these welcome efforts will achieve the full potential of the ITN method for those vulnerable people, which requires coverage of as many as possible of beds in the community with insecticidal nets, so as to maximize mosquito mortality.

## Competing interests

The author(s) declare that they have no competing interests.

## Authors' contributions

CAM, TR and SMM organised data collection and entry of it into a computer and they read and approved the manuscript. CFC analysed the data and drafted the manuscript.

## References

[B1] Lines JD, Myamba J, Curtis CF (1987). Experimental hut trials of permethrin impregnated mosquito nets and eave curtains against malaria vectors in Tanzania. Med Vet Ent.

[B2] Curtis CF, Myamba J, Wilkes TJ (1996). Comparison of different insecticides and fabrics for anti-mosquito bednets and curtains. Med Vet Ent.

[B3] Soremekun S, Maxwell CA, Zuwakuo M, Chen C, Michael E, Curtis CF (2004). Measuring the efficacy of insecticide treated bednets: the use of DNA fingerprinting to increase the accuracy of personal protection estimates in Tanzania. Trop Med Int Health.

[B4] Magesa SM, Wilkes TJ, Mnzava AEP, Njunwa KJ, Myamba J, Kivuyo MDP, Hill N, Lines JD, Curtis CF (1991). Trial of pyrethroid impregnated bednets in an area of Tanzania holoendemic for malaria, part 2: Effects on the malaria vector population. Acta Trop.

[B5] Maxwell CA, Myamba J, Njunwa KJ, Greenwood BM, Curtis CF (1999). Comparisons of bednets impregnated with different pyrethroids for their impact on mosquitoes and on re-infection with malaria after clearance of existing infections with chlorproguanil-dapsone. Trans R Soc Trop Med Hyg.

[B6] Maxwell CA, Chambo W, Mwaimu M, Magogo F, Carneiro IA, Curtis CF (2003). Variations in malaria transmission and morbidity with altitude in Tanzania and with introduction of alphacypermethrin treated nets. Malar J.

[B7] Schellenberg JR, Abdulla S, Nathan R, Mukasa O, Marchant TJ, Kikumbih N, Mushi AK, Mponda H, Minja H, Mshinda H, Tanner M, Lengeler C (2001). Effect of large scale social-marketing of insecticide-treated nets on child survival in rural Tanzania. Lancet.

[B8] Magesa SM, Lengeler C, de Savigny D, Miller JE, Njau RJA, Kramer K, Kitua A, Mwita A (2005). Creating an "enabling environment" for taking insecticide treated nets to national scale: the Tanzanian experience. Malar J.

[B9] Trape JF, Zoulani A (1987). Malaria and urbanization in Central Africa: the example of Brazzaville: III. Relationships between urbanization and the intensity of malaria transmission. Trans R Soc Trop Med Hyg.

[B10] Maxwell CA, Msuya E, Sudi M, Njunwa KJ, Carneiro IA, Curtis CF (2002). Effect on malaria morbidity of community-wide use in Tanzania of insecticide treated nets for 3–4 years. Trop Med Int Health.

[B11] Curtis CF, Maxwell CA, Finch RJ, Njunwa KJ (1998). A comparison of use of a pyrethroid either for house spraying or for bednet treatment against malaria vectors. Trop Med Int Health.

[B12] Curtis CF, Jana-Kara B, Maxwell CA (2003). Insecticide treated nets: impact on vector populations and relevance of initial intensity of transmission and pyrethroid resistance. J Vector Borne Diseases.

[B13] Maxwell CA, Myamba J, Magoma J, Rwegoshora RT, Magesa SM, Curtis CF (2006). Tests of Olyset nets by bioassay and in experimental huts. J Vector Borne Diseases.

[B14] Tami A, Mubyazi G, Talbert A, Mshinda H, Duchon S, Lengeler C (2004). Evaluation of Olyset insecticide-treated nets distributed seven years ago in Tanzania. Malar J.

[B15] Curtis CF, Maxwell C, Lemnge M, Kilama WL, Steketee RW, Hawley WA, Bergevin Y, Campbell CC, Sachs J, Teklehaimanot A, Ochola S, Guyatt H, Snow RW (2003). Scaling-up coverage with insecticide-treated nets against malaria in Africa: who should pay?. Lancet Inf Dis.

[B16] Erlanger TE, Enyati AA, Hemingway J, Mshinda H, Tami A, Lengeler C (2004). Field issues related to effectiveness of insecticide-treated nets in Tanzania. Med Vet Ent.

[B17] Grabowsky M, Farrell N, Hawley W, Chimumbwa J, Hoyer S, Wolkon A, Selaniko J (2005). Integrating insecticide-treated bednets into measles vaccination campaign achieves high, rapid and equitable coverage with direct and voucher-based methods. Trop Med Int Health.

